# Complex Regulation of *cyp26a1* Creates a Robust Retinoic Acid Gradient in the Zebrafish Embryo

**DOI:** 10.1371/journal.pbio.0050304

**Published:** 2007-11-20

**Authors:** Richard J White, Qing Nie, Arthur D Lander, Thomas F Schilling

**Affiliations:** 1 Department of Developmental and Cell Biology, University of California Irvine, Irvine, California, United States of America; 2 Department of Mathematics, University of California Irvine, Irvine, California, United States of America; 3 Center for Complex Biological Systems, University of California Irvine, Irvine, California, United States of America; 4 Developmental Biology Center, University of California Irvine, Irvine, California, United States of America; Wellcome Trust Sanger Institute, United Kingdom

## Abstract

Positional identities along the anterior–posterior axis of the vertebrate nervous system are assigned during gastrulation by multiple posteriorizing signals, including retinoic acid (RA), fibroblast growth factors (Fgfs), and Wnts. Experimental evidence has suggested that RA, which is produced in paraxial mesoderm posterior to the hindbrain by aldehyde dehydrogenase 1a2 (*aldh1a2*/*raldh2*), forms a posterior-to-anterior gradient across the hindbrain field, and provides the positional information that specifies the locations and fates of rhombomeres. Recently, alternative models have been proposed in which RA plays only a permissive role, signaling wherever it is not degraded. Here we use a combination of experimental and modeling tools to address the role of RA in providing long-range positional cues in the zebrafish hindbrain. Using cell transplantation and implantation of RA-coated beads into RA-deficient zebrafish embryos, we demonstrate that RA can directly convey graded positional information over long distances. We also show that expression of Cyp26a1, the major RA-degrading enzyme during gastrulation, is under complex feedback and feedforward control by RA and Fgf signaling. The predicted consequence of such control is that RA gradients will be both robust to fluctuations in RA synthesis and adaptive to changes in embryo length during gastrulation. Such control also provides an explanation for the fact that loss of an endogenous RA gradient can be compensated for by RA that is provided in a spatially uniform manner.

## Author Summary

The formation of gradients of morphogens, signaling molecules that determine cell fates in a concentration-dependent manner, is a fundamental process in developmental biology. Several morphogens pattern the anterior–posterior (head to tail) axis of the vertebrate nervous system, including the vitamin A derivative, retinoic acid (RA) and fibroblast growth factors (Fgfs). However, it remains unclear how the activities of such morphogen gradients are coordinated. We have addressed this question by combining genetic experiments in zebrafish and computational analyses. We show that RA acts as a graded signal over long distances and that its gradient is shaped, to a large extent, by local control of RA degradation. In particular, RA promotes and Fgf suppresses RA degradation, thereby linking the shapes of RA and Fgf gradients. Computational models suggest that this linkage helps make RA-mediated patterning robust to changes in the rate at which RA is synthesized (which may vary with levels of dietary vitamin A) as well as in the size and shape of the embryo during development. Analogous regulatory loops may be used for similar purposes in other tissues in which RA and Fgfs interact, as well as in other morphogen systems.

## Abstract 3

Experimental and computational studies in zebrafish reveal a complex system regulating degradation of the vitamin A derivative retinoic acid along the anterior-posterior axis, which helps explain how morphogen gradients are established and maintained.

## Introduction

The anterior–posterior (A–P) axis of vertebrate embryos is patterned by multiple signals. In the gastrula-stage embryo, at least three types of diffusible, extracellular molecules—retinoic acid (RA), fibroblast growth factors (Fgfs), and Wnts—together promote posterior and suppress anterior fates [1–6]. There is evidence for both hierarchical and parallel relationships among their signaling pathways. For example, RA appears to act downstream of Fgfs and Wnts in promoting posterior identities in the neurectoderm, but not in the suppression of anterior cell fates [6].

RA, Fgfs, and Wnts are all produced at the posterior of the embryo, and might therefore be expected to form posterior-to-anterior gradients (for Fgf8 this has been demonstrated directly; [7]). It is not clear which of these gradients is ultimately responsible for providing the positional cues that specify where cell fate boundaries form. This is an important question because the signaling pathways that drive expression of cell fates need not be the same as those that convey positional information. For example, it has recently been suggested that RA acts solely as a permissive factor in hindbrain patterning, required for the adoption of posterior fates, but downstream of signals that determine where such fates are specified [8]. Support for this view comes largely from observations that, in RA-deficient mouse, rat, quail, or zebrafish embryos, hindbrain pattern can be rescued by exposure of the embryo to uniform extracellular concentrations of RA [8–15].

Despite such observations, other experiments point to a role for RA that is more like that of a classical, graded morphogen. For example, embryos deficient in vitamin A (the dietary precursor of RA), or loss-of-function mutants in the RA synthetic enzyme aldehyde dehydrogenase 1a2 (*aldh1a2,* also known as *raldh2*), show loss of posterior hindbrain segments (rhombomeres) 5–7 (r5–7), and expansion of more anterior ones (r2–4) [9–11,15,16]. Treatment of chick embryos with an RA receptor (RAR) antagonist also causes progressive, concentration-dependent anteriorization [17]. Conversely, exposure of embryos to exogenous RA leads to a concentration-dependent loss of the forebrain and eyes, and progressive posteriorization of (rhombomere) identities [12,18–21]. Loss of *cyp26a1*—a cytochrome p450 enzyme that oxidizes RA to more polar metabolites, promoting its removal from tissues—causes r5–7 to expand anteriorly in mice and zebrafish [22–24], and the combined depletion of Cyp26a1 and related enzymes Cyp26b1 and Cyp26c1 results in severe posteriorization of the zebrafish hindbrain [8].

Efforts to understand the role of RA in hindbrain patterning have recently been fostered by the recognition that localized degradation is critical in controlling RA signaling. Sirbu et al. [25] suggested that degradation creates shifting boundaries of RA activity and that duration of exposure to RA rather than concentration controls rhombomere fate. In contrast, Maves et al. [12] argued that pattern is determined by an RA morphogen gradient that is not fixed, but grows steeper with time, specifying rhombomeres sequentially from anterior to posterior. Recently, Hernandez et al. [8] proposed a “gradient-free” model in which discrete zones of local RA degradation are progressively induced over time, in coordination with changes in cell responsiveness to RA. Since this model does not explain how such zones are placed in their correct positions, it reverts to an essentially permissive role for RA (i.e., positional information must still come from signals other than RA, such as Fgfs or Wnts).

Here we propose a new view that reconciles both existing and new data. In it, local degradation of RA plays a central role, yet long-range RA gradients directly provide graded positional cues. This model is motivated by experimental data on the control of expression of Cyp26 enzymes, and by computational analysis of feedback and feedforward interactions between RA and Fgf signaling that are revealed by these data. We argue that this makes the RA gradient resistant to fluctuations (robust) in RA synthesis rate and stable over an expanding field of cells. We also show that one characteristic of such a morphogen gradient system based on regulated degradation is the ability to produce gradients of relatively normal shape even when RA is applied globally to an embryo. The model also suggests a mechanism by which the incorporation of retinoids into patterning systems may have taken place during chordate evolution.

## Results

### Long-Range, Graded Effects of RA in Zebrafish Embryos

**Figure 1:**
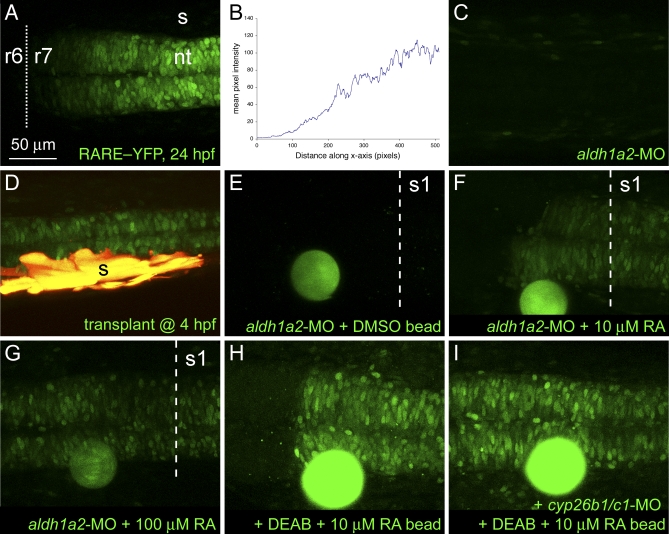
Responses of Embryos to Sources of RA. Long-range induction of a *rare:yfp* reporter. Confocal images of live embryos, dorsal view, anterior to the left. (A**)** *rare:yfp* expression in posterior hindbrain and spinal cord at 24 hpf with an anterior boundary at r6/7. (B) Quantification of fluorescence intensities in (A). (C) Lack of YFP expression in an *aldh1a2* morphant embryo. (D) Rescue of YFP expression in a morphant by somitic mesoderm (yellow) transplanted during gastrulation. (E–I) Bead implantation. RA-coated beads were implanted anterior to the first somite at 18–19 hpf and imaged at 23–24 hpf. A DMSO-coated bead (E) failed to rescue YFP expression, whereas beads soaked in either 10 μM (F) or 100 μM (G) RA induced YFP at distances of up to 200 μm. (H and I) Inhibition of both *cyp26b1* and *cyp26c1* in DEAB-treated embryos leads to a symmetrical response from the *rare:yfp* reporter to a bead soaked in 10 μM RA (compare [H] with [I]). The dotted line indicates the r6/7 boundary, the dashed line, the anterior border of somite 1. nt, neural tube; s, somites.

Studies in a variety of vertebrate embryos support the view that RA diffuses through tissues [26], can act at long range, and has graded effects on patterning [27–29]. However, direct evidence that diffusible RA sets up a gradient of RA response, which in turn assigns different fates to cells at different levels of RA signaling, is still lacking. To investigate this question, we utilized a zebrafish line carrying a *yellow fluorescent protein* (*yfp)* transgene under the control of retinoic acid response elements (*rare:yfp* [30]). In transgenic embryos, expression of YFP was readily detectable in the developing spinal cord and posterior hindbrain at 22–24 hours post-fertilization (hpf) (up to the r6/r7 boundary; Figure 1A), and was clearly graded from posterior to anterior in the posterior hindbrain (Figure 1B). Expression was completely lost in RA-deficient embryos injected with an *aldh1a2* morpholino (*aldh1a2*-MO; Figure 1C), and could be restored by the prior transplantation of cells overexpressing *aldh1a2* and targeted to somitic mesoderm, where *aldh1a2* is normally expressed (Figure 1D). In most cases, rescue spanned the width of the neural tube (approximately six cell diameters [∼70 μm]; Table 1).

**Table 1:**
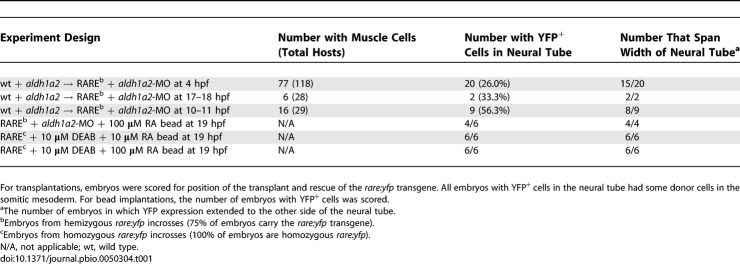
Summary of Transplants and Bead Implantations

Similarly, ion-exchange beads soaked in 10–100 μM RA and implanted just anterior to the first somite at 19 hpf also strongly induced *rare:yfp* (Figure 1E–1G). YFP expression was observed at a greater distance—up to 300 μm—when beads were soaked in 100 μM RA (Table 1). Interestingly, responses to low (10 μM) doses of RA were markedly asymmetrical (Figure 1F and 1H), extending up to 300 μm posteriorly but truncated anteriorly near the r6/7 boundary. This asymmetry was eliminated with MOs directed against *cyp26b1* and *cyp26c1* (Figure 1I), which are highly expressed just anterior to the r6/7 boundary [8].

From these results, we infer that RA signaling in the neural ectoderm is graded in vivo, that signaling is dose dependent, and that long-range effects of RA can be mediated across territories in which RA cannot be synthesized (ruling out a relay-type model of RA action at a distance). In addition, asymmetries in the response to RA signaling arise in a manner consistent with localized effects of Cyp26 enzymes on RA responsiveness, as previously proposed [8]

**Figure 2:**
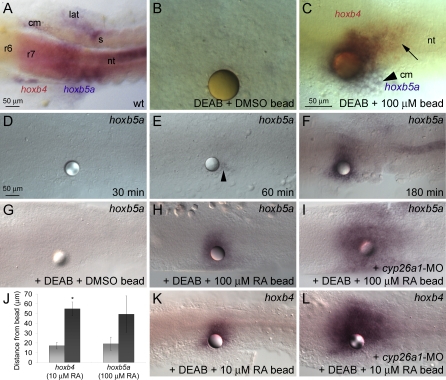
Concentration-Dependent Induction of *hox* Gene Expression by RA (A) In wild-type (wt) embryos, *hoxb4* expression in the neural tube (nt) extends to the r6/7 boundary, whereas the limit of *hoxb5a* expression is level with the first somite (s). *hoxb5a* is also expressed in axial, lateral (lat), and cranial (cm) mesoderm. (B and C) Beads soaked in 100 μM RA were implanted at 1–3 somites (10.3–11 hpf) into DEAB-treated embryos, fixed 3 h later, and assayed for *hox* expression. Dorsal views, showing in situ hybridization with probes for *hoxb4* and *hoxb5a* at 14 hpf. (B) DMSO-coated beads do not induce *hox* expression. (C) RA-coated beads induce *hoxb5a* in lateral mesoderm (arrowhead), *hoxb5a*/*hoxb4* double-positive cells near the bead, and *hoxb4* single-positive cells further away (black arrow). (D–F) Time-course of *hoxb5a* induction in response to a 100 μM RA bead: no induction 30 min post-implantation** (**D**)**, a few cells near the bead after 60 min (arrowhead) (E**),** and strong expression further away after 180 min (F). (G–L) RA-induced degradation limits the range of RA signaling. RA beads were implanted at 1–3 somites (10.3–11 hpf) into DEAB-treated embryos, fixed 3 h later, and assayed for *hox* expression. Dorsal views, showing in situ hybridization with probes for *hoxb5a* (G–I) or *hoxb4* (K and L) at 14 hpf. (G) Implantation of a DMSO bead has no effect on gene expression in DEAB–treated embryos. (H and I) A 100 μM RA bead placed into a DEAB-treated, *cyp26a1* morphant (I) induces *hoxb5a* over a much larger area than a DEAB-treated control (H). (J) Graph showing an increase in the range of *hox* induction in *cyp26a1* morphant, DEAB-treated embryos (black bar) as compared to DEAB-treated (light grey bar). The asterisk (*) indicates significantly different from DEAB-treated controls (*p* < 0.05), using the Student *t*-test. (K and L) A 10 μM RA bead placed into a *cyp26a1* morphant induces *hoxb4* over a much larger area.

The *rare:yfp* reporter appears primarily to read out sustained, high-level RA signaling. For example, YFP was not normally detected in transgenic gastrula- or early somite-stage embryos, when RA is known to act, but could be induced by beads soaked in very high levels of RA (10–25mM; Figure S1A–S1C). As a more sensitive readout of early RA signaling, we monitored two direct RA target genes, *hoxb4* and *hoxb5a* [31]. Normally, *hoxb4* has an anterior limit at the r6/7 boundary, whereas *hoxb5a* is expressed further posteriorly in the spinal cord (Figure 2A). Both genes require RA signaling for their early expression (Figure 2B) and both possess functional, phylogenetically conserved RAREs within their promoters [32,33]. Since *hoxb4* is expressed more anteriorly, one might expect it to be activated by lower RA concentrations than *hoxb5a*. Consistent with this, beads coated in 100 μM RA and implanted into embryos treated with 10 μM diethylaminobenzaldehyde ([DEAB), which inhibits class 1 aldehyde dehydrogenases and thereby blocks RA synthesis [12,14,34,35], induced expression of both *hox* genes (Figure 2C) at early neurula stages (10–11 hpf). Induction was detectable within 60 min of bead implantation (Figure 2D–2F), and consistently extended further from the bead for *hoxb4* (94.86 ± 17.18 μm; *n* = 4) than for *hoxb5a* (38.44 ± 6.27 μm; *n* = 5). These results suggest that there are multiple thresholds of RA response, with more anteriorly expressed target genes exhibiting lower expression thresholds. Together, the results in Figures 1 and 2 imply that RA can establish long-range gradients that directly specify the location of expression of genes that control A–P identity.

### Effects of RA-Induced Feedback on RA Degradation and the Range of RA Signaling

**Figure 3:**
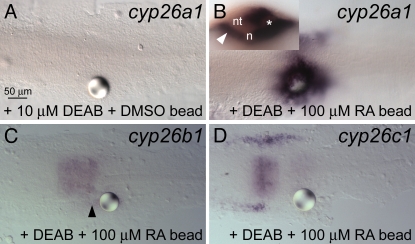
Regulation of Cyp26s by RA (A) Loss of *cyp26a1* expression in cranial mesoderm in DEAB-treated embryos and lack of induction by a DMSO-soaked bead. (B) Induction of *cyp26a1* expression by an RA bead; inset, optical section showing *cyp26a1* induction in both the neural tube (nt), lying above the notochord (n), and lateral mesoderm (white arrowhead). The asterisk (*) indicates the bead. (C) Weak induction of *cyp26b1* (black arrowhead). (D) No induction of *cyp26c1*.

In contrast to the loss of asymmetry in the RA response at 24 hpf caused by reducing *cyp26b1* and *cyp26c1* function (Figure 1H and 1I), the range of *hoxb4* and *hoxb5a* responses at 13–14 hpf was more than doubled by inhibiting *cyp26a1* function (Figure 2G–2L). This was unexpected because, at the stage of bead implantation for these experiments, *cyp26a1* is not normally expressed within the hindbrain (Figure 3A). This suggested that RA beads were themselves inducing *cyp26a1*. Indeed, in situ hybridization revealed marked *cyp26a1* induction up to 53 μm from the bead (Figure 3B). In contrast, RA beads only weakly induced *cyp26b1* expression (in cranial mesoderm; Figure 3C, arrowhead), and did not induce *cyp26c1* at all (Figure 3D), findings that are consistent with studies in chick [36]. Whole-embryo treatment with high RA levels (1 μM) also failed to induce *cyp26b1* or *cyp26c1*, suggesting that these enzymes do not simply have lower sensitivities to RA than *cyp26a1* (unpublished data). If anything, RA appeared to have a short-range inhibitory effect on *cyp26c1* expression (Figure 3D).

Although it has been observed that exogenous RA induces *cyp26a1* in the hindbrain [36,37], loss of RA signaling does not affect endogenous *cyp26a1* expression [8,25,37]. This has led to the view that *cyp26a1* induction occurs only at supra-physiological levels of RA, possibly as a form of protection against teratogenicity [8]. Yet in Figure 3, the range over which *cyp26a1* is induced implies that its expression is triggered by levels of RA similar to those that induce *hoxb4* and *hoxb5a*. This result is even more curious in view of the fact that these *hox* genes and *cyp26a1* are not normally coexpressed in the neurectoderm.

**Figure 4:**
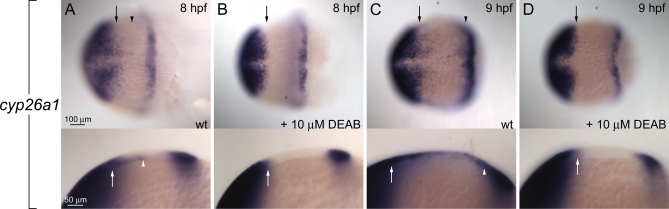
RA-Dependent Expression of *cyp26a1* Wild-type (wt) and DEAB-treated embryos were stained for extended periods of time for *cyp26a1* expression to investigate low-level expression. Top, dorsal view; bottom, lateral view. (A and C) Weak expression posterior to the presumptive midbrain (arrow to arrowhead). (B and D) This expression is lost in DEAB-treated embryos.

To try to make sense of these observations, we reexamined endogenous patterns of *cyp26a1* expression in embryos, starting at gastrula stage (4–9 hpf; Figure 4). At this stage, *cyp26a1* is reported to be restricted anteriorly to the animal pole in the presumptive forebrain and midbrain, and posteriorly to the involuting margin [6,8]. Close inspection, however, revealed the presence of previously unrecognized, low-level *cyp26a1* expression in between these two sites (Figure 4A and 4C). This was observed at mid-gastrula stages (75% epiboly), predominantly in the hypoblast (future mesendoderm; lower panel, Figure 4A) and extended approximately halfway between the anterior and marginal *cyp26a1* expression domains (arrow to arrowhead on Figure 4A). By late gastrula (90% epiboly), weak *cyp26a1* expression covered the entire region between the anterior and marginal domains (arrow to arrowhead on Figure 4C) and was observed in both the epiblast (future ectoderm) and hypoblast (lower panel, Figure 4C), encompassing the entire presumptive hindbrain field. Because this expression was weak, it might easily have been overlooked were it not for the fact that it completely disappeared upon treatment with 10 μM DEAB (Figure 4B and 4D). In contrast (and as previously reported), the strong expression of *cyp26a1* in the anterior central nervous system and posterior margin was RA-independent [37]. Similar RA-dependent, weak *cyp26a1* expression in the presumptive hindbrain can be seen in a recent study of vitamin A–deficient quails (see Figure 3B of [36]).

**Figure 5:**
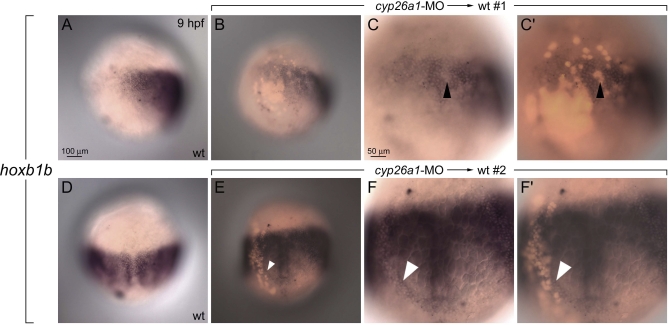
*cyp26a1* Is Required Outside of the Anterior Neural Domain (A and D) *hoxb1b* expression in wild-type (wt) embryos. (A–C′) Lateral view, anterior to top, dorsal to right. (D–F′) Dorsal view, anterior to top. (B, C′, E, and F′) Merged brightfield and fluorescent images show positions of transplanted cyp26a1-deficient cells labeled with lysine-fixable rhodamine dextran (red). (B–C′) Transplants of cyp26a1-deficient cells located within the endogenous *hoxb1b* domain cause a nearly cell-autonomous decrease in *hoxb1b* expression (black arrowheads). (E–F′) Transplants of cyp26a1-deficient cells also cause changes in *hoxb1b* expression in adjacent host cells (white arrowheads).

These data indicate that expression of the major RA-degrading enzyme, *cyp26a1*, in cells within or adjacent to the presumptive hindbrain during gastrulation is under the control of RA signaling. This suggests a more subtle role for RA degradation than simply creating boundaries beyond which RA cannot spread. Given that expression at such locations is weak, the question arises as to whether it is of any physiological consequence. To address this, we used mosaic analysis, transplanting *cyp26a1* morphant cells at 5 hpf (50% epiboly) into wild-type embryos and assaying the expression of *hoxb1b* at 9–10 hpf (90% epiboly–tailbud; Figure 5A and 5D). When located within the endogenous *hoxb1b* domain, Cyp26a1-deficient cells down-regulated *hoxb1b* (Figure 5B–5C′), Cyp26a1-deficient cells also often caused an increase in expression in adjacent cells (Figure 5E–5F′). These results indicate that the low-level expression of *cyp26a1* within and near the presumptive hindbrain is, indeed, functionally relevant. Moreover, because the observed effects were limited either to the transplanted cells themselves or cells nearby, the results imply that *cyp26a1* normally influences RA signaling only over very short ranges (the significance of this point will be discussed later).

### Fgf Regulates *cyp26a1* Expression

If low-level *cyp26a1* expression in the future hindbrain of the gastrula-stage embryo is induced by RA signaling, why does such expression decline from anterior to posterior (Figure 4A–4D), a direction along which RA signaling presumably increases? This observation suggests that there must be additional, position-specific inputs into *cyp26a1*.

**Figure 6:**
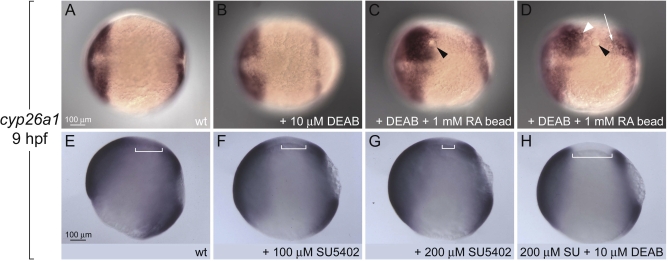
Regulation of *cyp26a1* by RA and Fgfs (A and B) Anterior and marginal expression domains of *cyp26a1* expression are unchanged by 10 μM DEAB. Anterior is to the left in all panels. (C and D) RA beads (black arrowheads) induce *cyp26a1* expression in DEAB-treated embryos far from (white arrowhead and arrow in [D]), but not adjacent to the bead. (E–H) Fgf signaling sets the posterior boundary of *cyp26a1* expression. (E) *cyp26a1* expression in wild type (wt) (lateral view, anterior to the left). (F and G) Expression in SU5402-treated embryos shifts posteriorly in a concentration-dependent manner (compare brackets in [E], [F] and [G]). (H) The posterior shift caused by blocking Fgf signaling is abolished by DEAB treatment.

To clarify the nature of such inputs, we implanted RA beads into different A–P locations in DEAB-treated embryos at mid-gastrula (8 hpf). This rapidly induced *cyp26a1* expression (≤30 min), but the pattern of induction depended on the precise bead position (Figure 6A–6D). Beads placed within 100 μm of the anterior domain of high-level *cyp26a1* expression in the brain induced *cyp26a1* anterior to the bead—in a pattern that merged with endogenous *cyp26a1*—but not posteriorly (Figure 6C). Beads placed further posteriorly at mid-trunk levels (Figure 6D), failed to induce *cyp26a1* expression near the bead at all (where RA levels should be highest), but only far anteriorly (white arrowhead) and near the posterior margin (white arrow). These results suggest that the capacity of cells to induce *cyp26a1* in response to RA declines from anterior to posterior, rising again near the posterior end of the embryo. A similar phenomenon can be seen if DEAB-treated embryos are treated with increasing concentrations of exogenous RA (Figure S2A–S2D).

An attractive candidate for a factor controlling such positional effects is Fgf. Eliminating all Fgf signaling is known to lead to *cyp26a1* expression along the entire A–P axis [6], which suggests that one role of Fgfs may be to suppress *cyp26a1* expression. We examined this possibility by treating embryos with various concentrations of the Fgf receptor tyrosine kinase inhibitor SU5402. As shown in Figure 6E–6G, in the presence of SU5402, the border of high-level *cyp26a1* in the neurectoderm shifted, in a concentration-dependent fashion, progressively toward the posterior. Interestingly, the expansion of *cyp26a1* expression upon blockade of Fgf signaling was prevented by simultaneous administration of DEAB (Figure 6H). Thus, Fgf appears to act indirectly, by inhibiting RA-mediated activation of *cyp26a1* expression.

### Modeling the RA Gradient System

From the information presented above, we infer that RA gradients, at least during gastrulation, should be influenced by interacting feedback (RA signaling inducing RA degradation) and feedforward (Fgf signaling repressing RA degradation) effects. To identify the consequences of such effects, we turned to quantitative modeling, using partial differential equations to describe the spatial dynamics of RA diffusion and degradation (see Materials and Methods). Because RA is a lipophilic molecule that can pass directly through cell membranes, we initially ignored cell boundaries, modeling RA as a substance that moves freely through tissue, being degraded at any location in proportion to the amount of Cyp26 enzyme expressed by cells there. However, the observations in Figure 5 made us realize that this could not be the case. The fact that the effects of blockade of *cyp26a1* in transplanted cells were nearly cell autonomous tells us that RA molecules that enter cells with even low levels of *cyp26a1* expression get degraded before they have an opportunity to exit the cell. The only way that RA can act over long distances, yet effectively never leave cells, is if transport precedes cell entry, i.e., RA moves a long way within the extracellular space before it is taken up.

In fact, this makes sense when one considers that RA binds proteins tightly, and must dissociate from them to cross a plasma membrane. It has been shown in cell cultures that the presence of physiological levels of albumin results in very slow RA uptake by cells [38]. Given that RA bound to a soluble protein carrier could be expected to diffuse several hundred micrometers in a matter of minutes, we reasoned that a realistic model of RA dynamics needs to include separate, interconverting pools of extracellular protein-bound and intracellular RA, with RA signaling being solely a function of the latter.

**Figure 7:**
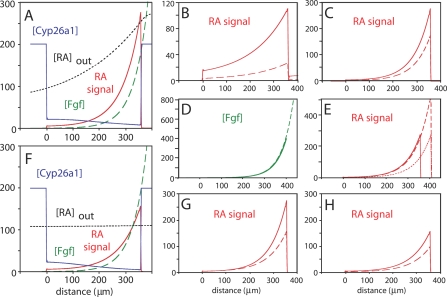
Modeling the RA Morphogen Gradient A one-dimensional mathematical model of the gastrula-stage zebrafish embryo was developed that incorporates RA synthesis, diffusion, cell permeation, degradation, and signaling; a stable Fgf gradient; and expression of *cyp26a1* under the control of RA and Fgf signaling, as well as other position-specific cues (see Materials and Methods). In each panel, anterior is to the left and posterior to the right. On the abscissa, zero represents the posterior boundary of the domain of high, anterior *cyp26a1* expression (approximately the r1/r2 border), which is set independently of RA. Values on the ordinate are in arbitrary units. Fgf concentration is shown in green, Cyp26a1 in blue, extracellular RA ([RA]*_out_*) in black, and RA signaling (a function of intracellular RA) is shown in red. Parameter values common to all panels are given in Materials and Methods. (A) Typical patterns of RA signaling and *cyp26a1* expression generated by the model. Note the presence of low-level *cyp26a1* expression that declines from anterior to posterior. (B and C) Feedback and feedforward regulation of *cyp26a1* expression leads to robustness to RA synthesis rates. In both panels, the dashed curves show the effects of a 2-fold decrease in RA production. In (B), *cyp26a1* is taken to be constant over the interval 0 < *x* < 360 μm, whereas in (C), it is regulated as prescribed by the model. Note the dramatic increase in robustness in (C). (D and E) Effect of embryo elongation on Fgf and RA gradients. In both panels, solid lines are calculated for a posterior margin at 400 μm; dashed lines for a posterior margin at 450 μm. In (D), the dashed line is calculated using a rate of Fgf synthesis 2.75 times higher than for the solid line; this is to the degree of increase that would be required to compensate for the rearward movement of the Fgf source. (E) shows that, using the same sets of parameters, the RA gradient automatically compensates for the rearward movement of the RA source, without any need for readjustment of RA synthesis. For comparison, the dotted line shows what would happen to the RA gradient were there no such compensation. (F and G) RA supplied in a completely delocalized manner can produce a relatively normal morphogen gradient. (F) is a modification of (A), in which the endogenous posterior source of RA (from 360 < *x* < 400) has replaced by a constant influx of RA at all locations. For an appropriate rate of exogenous RA influx, relatively normal patterns of RA signaling and *cyp26a1* expression ensue, despite the lack of an extracellular RA gradient. This is particularly true at anterior locations, as shown in (G), in which the RA signaling gradients from (A) and (F) are compared (solid and dashed curves, respectively). (H) RA signaling gradients produced by delocalized RA are robust to rates of RA influx. Like gradients produced by endogenous RA (C), gradients produced by exogenous, delocalized RA are also predicted to be robust (solid curve is RA signaling from [F]; dashed curve shows the effect of a 2-fold decrease the in the rate of RA influx). Values of parameters that differed between panels were β = 1 ([A–E] and solid curve in [G]) or 0.5 ([F], [H], and dashed curve in [G]); *x*_f_ = 400 μm ([A–C] and [F–H]) or 450 μm (D–E); *f*_0_ = 0 (B), 400 ([A], [C], [F–H], and solid curves in [D] and [E]), or 1,100 (dashed curves in [D] and [E]); *k*_deg_ = 500 sec^−1^ ([A] and [C–G]) or 0.03 sec^−1^ (B); *k*_max_ = 0.001 ([A] and [C–G]) or 4 (B); *V*(*x*) = 5 for *x* > *x*_f_ – 40, zero otherwise ([A], [D], [E], and solid curves in [B], [C], and [G]), 2.5 for *x* > *x*_f_ – 40, zero otherwise (dashed curves in [B] and [C]), 0.2 for all *x* ([F], dashed curve in [G], and solid curve in [H]), or 0.1 for all *x* (dashed curve in [H]).

We developed such a model for the early gastrula-stage embryo (see Materials and Methods), when RA is known to be required for hindbrain patterning, and its behavior was analyzed over a wide range of parameter choices (Figure 7). The model readily generates patterns of RA signaling and *cyp26a1* expression (Figure 7A) that, for reasonable parameter choices, fit experimental expectations. As a result of exploration of the model, three observations were made:

First, feedback regulation of RA degradation makes the intracellular RA gradient robust to changes in the rate of RA synthesis. In the absence of any controls on *cyp26a1* expression, a change in the rate of RA synthesis would be expected to produce a similar change in the amplitude of the RA gradient. Figure 7B illustrates the effect of a 2-fold decrease in RA synthesis rate on the (intracellular) RA signaling gradient. Note the large shift in the locations at which RA signaling levels cross threshold values. In contrast, by allowing for strong feedback regulation of *cyp26a1*, the model produces a much more robust gradient (Figure 7C). The idea that “self-enhanced degradation” could render morphogen gradients robust to fluctuations in synthesis rates was first shown in theoretical work on Wingless and Hedgehog gradients in *Drosophila* [39]. Since rates of RA synthesis in vivo are likely to be highly labile to environmental factors (e.g., the availability of its dietary precursor, vitamin A), exploitation of such a mechanism would seem especially advantageous for RA gradients.

Second, feedforward effects of Fgfs couple the shape of the RA gradient to that of the Fgf gradient. Previous work has shown that the Fgf gradient is formed by diffusion and receptor-mediated destruction [7], so its shape should be determined by a balance between Fgf synthesis and receptor binding and dynamics [40,41]. During gastrulation, the source of Fgf expression in the posterior marginal zone moves continuously away from the anterior of the embryo, so the Fgf gradient itself would need to undergo continual change in order to maintain stable levels of Fgf at constant positions. The easiest way to accomplish this would be for Fgf expression to increase as gastrulation proceeds (Figure 7D). Were the RA gradient not coupled to the Fgf gradient through the latter's effects on RA degradation, elongation of the embryo would continually shift the RA gradient toward the posterior (Figure 7E, dotted line), unless RA synthesis were continually increased by just the right amount. In contrast, because of Fgf's influence on *cyp26a1* induction, increased Fgf production automatically results in an increase in RA signaling that compensates for axis elongation (Figure 7E, dashed line). In effect, the RA gradient becomes entrained to the Fgf gradient.

Third, even an embryo that makes no RA and is bathed in uniform RA will generate a relatively normal RA signaling gradient. A remarkable consequence of the entrainment of the RA gradient to the Fgf gradient is that replacement of the endogenous RA source at the posterior with a spatially uniform influx of RA into the extracellular space still produces a marked posterior-to-anterior gradient of intracellular RA (Figure 7F). At appropriate doses of exogenous RA, the RA signaling gradient will closely resemble the endogenous one, especially at anterior sites (Figure 7G). This effect nicely reconciles data showing that RA acts in a concentration-dependent manner [12,17–21] with observations that uniform administration of RA can effectively rescue pattern in RA-deficient embryos [8–12].

Furthermore, because of the feedback effect of RA on its own degradation, the exogenous gradient can be expected to be as robust to RA dosage as the endogenous one (compare Figure 7H and 7C). Indeed, marked robustness of rescue by exogenous RA can be demonstrated experimentally (Figure S3): when DEAB-treated embryos were exposed to exogenous RA starting at 4 hpf, relatively normal hindbrain patterning was restored over an 8-fold range (0.625–5 nM) of RA concentrations. Hernandez et al. [8] reported similar rescue over a 20-fold RA range (0.5–10 nM), using embryos treated at slightly later stages (5.3 hpf). We interpret such robustness not as an indication that RA acts in a concentration-independent manner, but rather that the distribution and concentration of RA within cells is controlled through RA- and Fgf-signaling–dependent control processes, such as those described here.

## Discussion

### RA and Fgf Collaborate to Produce a Robust Patterning System

**Figure 8:**
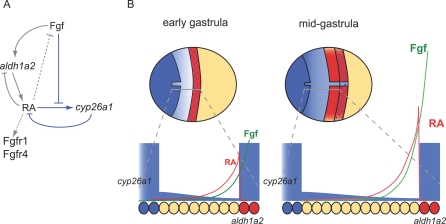
Model of Hindbrain Patterning by RA (A) Diagram of interactions between RA, Fgf, and *cyp26a1*. Only those shown in blue are included in the mathematical model; dotted lines are extrapolated from other animal models. (B) Schematics illustrate dorsal views of gastrulating embryos and corresponding gradients, anterior to the left. RA is produced by Aldh1a2 in somitic mesoderm (red) and diffuses through the neurectoderm. Cyp26a1 (blue) degrades RA at differing rates to produce a gradient across the hindbrain that specifies rhombomere fates. The regulation of *cyp26a1* by both Fgf and RA signaling creates a robust RA gradient that grows appropriately as the A–P axis of the embryo elongates.

The classical view of a morphogen is a molecule with a graded distribution that acts in a concentration-dependent fashion to assign positional identities to a field of cells [42]. The question of whether RA is a morphogen has been controversial in a number of contexts, including patterning of the limb, heart, and brain [8,12,43–47]. Because RA so often acts together with other signaling molecules—such as Fgfs, Hedgehogs, and Bmps—to influence patterning, the issue of whether RA plays an instructive or merely permissive role in conveying positional information is a continual source of debate. Here, we suggest that the key to resolving this debate—in the hindbrain at least—is to treat RA and Fgf as a single, integrated morphogen system, in which an RA gradient assigns positional identities, but the shape of the RA gradient is established collaboratively by RA and Fgf through their control of *cyp26a1* expression (Figure 8).

This view reconciles several observations: It agrees with experimental evidence that RA and Fgf both promote posterior identity, with RA acting downstream of Fgf [6]. It accommodates data that RA acts over long range to produce graded outputs (Figures 1 and 2), and that both Fgf and RA control the distribution of RA-degradative enzymes (Figures 3–5). Perhaps most importantly, it explains how administration of uniform, exogenous RA can rescue patterning that is normally driven by RA synthesized in a localized fashion (Figure 7). This view also provides a mechanism by which the RA gradient can grow over time during gastrulation (Figure 8B)—a key element of the hindbrain patterning model of Maves and Kimmel [12]—without the need for complex regulation of RA synthesis. Although this still requires Fgf signaling to increase during gastrulation, the known coupling of Fgf signaling to gastrulation movements provides a means by which such increases may be coordinated [48,49].

Central to the mechanism proposed in this study is the regulation of RA degradation in a manner that provides for robustness to uncertain levels of RA synthesis. Evidence that RA synthesis is highly susceptible to fluctuations in levels of dietary precursors includes the fact that high doses of vitamin A readily produce retinoid teratogenicity in mammals [50]. Moreover, in the zebrafish embryo, even modest increases in intracellular levels of the RA precursor retinal lead to strong posteriorization if Cyp26a1 function is blocked [8]. Although the latter effect has been interpreted as evidence that Cyp26a1 exists to protect embryos from teratogenicity, it is also a consequence of our model in which Cyp26a1 plays a central role in controlling the shape of an RA gradient. Surprisingly, this role is not one in which Cyp26a1 simply acts as a sink for extracellular RA, since removing Cyp26a1 function in transplanted cells has nearly cell autonomous effects (Figure 5). This suggests that RA degradation within a cell does not greatly affect the concentration of RA within a neighboring cell and that RA must act over long distances by traveling within the extracellular space before it enters cells.

Indeed, our results agree with those of Sirbu et al. [25], Hernandez et al. [8], and others in emphasizing the importance of localized degradation in controlling RA signaling in the developing hindbrain. Unlike Hernandez et al. [8], however, we find no need to reject the idea of an RA gradient in favor of a model driven by the sequential appearance of sharp boundaries of *cyp26b1* and *cyp26c1* expression. In fact, an RA gradient primarily controlled by early, smoothly graded Cyp26a1 activity better explains the relatively mild hindbrain phenotypes of *cyp26b1*/*cyp26c1* double morphants, and the requirement for *cyp26a1* to be functional for global RA treatments to rescue RA-deficient embryos.

### Towards a More Complete Understanding of RA Patterning Systems

In the present study, a mathematical model was used to explore the consequences of feedforward and feedback effects of Fgf and RA on hindbrain patterning. Such a model is not intended to represent a complete or accurate picture of the early embryo, but simply to elucidate how interacting signaling pathways might work together to achieve useful ends. In fact, there is evidence that the regulatory interactions in this system go well beyond those explicitly modeled here (Figure 8A). For example, there is evidence that RA signaling inhibits expression of the RA biosynthetic enzyme *aldh1a2* [37], which is likely to further decrease the sensitivity to RA synthesis to RA precursor levels. RA may affect the expression of Fgfs, and Fgf signaling is required for the expression of *aldh1a2* [51]. RA signaling also appears to have complex effects on the expression of Fgf receptors [51]. Determining whether these effects contribute to robustness, to the coupling between RA and Fgf gradients, or to the performance of other tasks, awaits further experimental and computational work.

It should also be noted that, whereas the present model treats the RA gradient as a system operating near its steady state, the rapidity with which gastrulation proceeds in the zebrafish raises the possibility that patterning occurs under pre–steady-state conditions. Interestingly, recent theoretical work suggests that responses to morphogen gradients in the pre–steady-state regime can be much more robust than those at steady state [52], and preliminary calculations confirm that this is true for the RA gradient model described here (unpublished data). Thus, the sources of robustness in RA gradients may be many and varied.

Collaboration between RA and Fgfs appears to be emerging as a common motif in vertebrate pattern formation, not only in the nervous system (e.g., hindbrain, forebrain, ventral retina, and tailbud), but elsewhere (e.g., somites, heart, pancreas, and limbs). Intriguingly, the details of the interactions between RA and Fgfs are often quite different in different systems. For example, RA and Fgf gradients with opposite, rather than parallel, orientation are thought to play key roles in the patterning of somites, and recent modeling studies have provided insights into the tasks such an arrangement can accomplish [53,54]. It will be interesting to see whether or not there are common regulatory loops and control mechanisms in all RA/Fgf systems. It will also be interesting to see whether or not analogous collaborative relationships exist among other morphogens that pattern animal embryos.

### Evolution of the RA Gradient System

The roles of Fgf and RA in A–P patterning appear to have arisen in the deuterostome lineage, as protostomes (e.g., nematodes and arthropods) apparently lack both RARs and the enzymes that synthesize RA [55]. Yet nuclear hormone receptors, including Retinoid X receptors (RXR)s, are expressed throughout the animal kingdom and serve in the detection of both endogenous hormones and environmental compounds [56,57]. Invertebrates also use cytochrome p450 enzymes to oxidize a wide variety of endogenous and environmental compounds [58,59]. Our results suggest that the initial step in the evolution of the RA gradient system may have occurred when one of these enzymes happened to fall under the control of an ancestral A–P patterning system, so that its expression in embryos became graded from posterior to anterior. As a result, any environmental substance that it degraded would automatically form a gradient within the embryo, the shape of which would be read out at the transcriptional level through RXR-mediated signaling. In this way, a primitive RA-like gradient system could be established that was later refined, e.g., by developing ways to store the precursor of the compound, and produce an active form of it at one end of the embryo.

Although we can only speculate as to the advantage gained by incorporating RA into a preexisting A–P patterning system, we note that Kerszberg [60] showed that gradients of nuclear hormone receptor ligands have an intrinsic ability to form domains of gene expression with multiple sharp boundaries. Bringing RA into A–P patterning may thus have been critical in the evolution of the highly subdivided, rhombomeric organization of the vertebrate hindbrain.

## Materials and Methods

### Animals.

Embryos were obtained in natural crosses and staged according to Kimmel et al. [61]. *rare:yfp* (Tg(RARE-gata2:NTD-eYFP)—ZDB-LOCUS-051209–1) fish [30] were a kind gift of E. Linney.

### Morpholinos.

Antisense MOs were designed to translation start sites in *cyp26b1* (5′-TCAAAACTCTCGAAGAGCATGGCTG-3′) and *cyp26c1/d1* (5′-AAATCGTGCCCGAACATCTCGAACG-3′). *aldh1a2*- and *cyp26a1*-MOs have been described [11,24]. MOs were dissolved in Danieau buffer; 0.5 nl of solution was injected at the one-cell stage.

### Cell transplantation.

*rare:yfp experiments.* Wild-type donor embryos were injected at the one-cell stage with a mixture of 1.5% TRITC-dextran (neutral, 10,000 *M*_r_), 1.5% biotinylated-dextran (lysine-fixable, 10,000 *M*_r_), and 2 ng of *aldh1a2* RNA, and treated with 10 nM retinol (vitamin A). *rare:yfp* host embryos were injected with 1 ng of *aldh1a2*-MO. At sphere stage, embryos were mounted in 3% methylcellulose, and 30–50 cells were transplanted from the margins of labeled donor embryos to the margins of unlabeled hosts. Post-gastrula transplants were performed by mounting embryos in 0.75% low-melt agarose, and cells were transplanted from the somites of donor embryos into the forming somites of host embryos. Transplanted embryos were mounted in 1.5% agarose for analysis by confocal microscopy (using a Zeiss LSM510Meta). Images were processed, and the fluorescence intensity graph was produced using ImageJ software (http://rsb.info.nih.gov/ij/).

*cyp26a1-MO experiments.* Wild-type donor embryos were injected at the one-cell stage with a mixture of 1.5% Fluor-ruby-dextran (lysine-fixable, 10,000 *M*_r_), 1.5% biotinylated-dextran (lysine-fixable, 10,000 *M*_r_), and 2 ng of *cyp26a1*-MO. At 30%–50% epiboly (4.7–5.3 hpf), 30–50 cells were transplanted from the margins of labeled donor embryos to the margins of unlabeled wild-type hosts. Transplanted embryos were fixed at 90% epiboly (9 hpf) and stained for *hoxb1b* expression by in situ hybridization.

### Pharmacological treatments.

The following stock solutions were stored in DMSO at −80 °C: 10 mM 4-(diethylamino)-benzaldehyde (DEAB; Acros organics), 10 mM all-trans retinoic acid (RA; Sigma), and 10 mM SU5402 (CalBiochem). These were diluted in Embryo Medium (EM) for treatments. Sibling controls were incubated in corresponding dilutions of DMSO. All incubations were done in the dark.

### Bead implantation.

AG 1-X8 ion-exchange beads (formate form; BIORAD) were soaked in different concentrations of all-trans RA in EM for 1 h (control beads were incubated in similar amounts of DMSO in EM). Beads were rinsed in EM for 5 min before implantation. Embryos were mounted in 3% methylcellulose, a small slit was cut in the epidermis with a tungsten needle, and an RA/DMSO-coated bead (40–60 μm in diameter) was inserted through the slit. Embryos were then incubated at 28.5 °C in the dark prior to fixation.

### Whole-mount in situ hybridization.

In situ hybridization was carried out as described previously [62]. Probes included *hoxb4*, *hoxb5a*, and *hoxd4a* [31], *cyp26a1* [6], *cyp26b1* (IMAGEclone3722563; EcoRI, SP6), *cyp26c1/d1* [63], *hoxb1b* [64], *krox-20/egr2b* [65], and *pax2a* [66] . Images were taken and measurements were made using Openlab software (http://www.improvision.com/products/openlab/). Ranges of *hox* gene induction (e.g., assayed as the distance from the edge of the bead to the most distant *hox*-expressing cell) were compared using a two-tailed Student *t*-test.

### Modeling.

The RA gradient in the gastrula was modeled as a one-dimensional reaction diffusion system (cf. [41,67,68]) represented by the following equations.

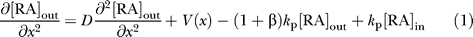


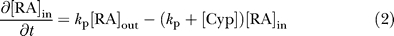


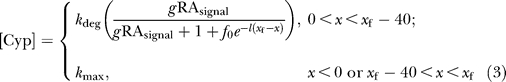


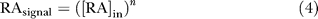



[RA]_out_ and [RA]_in_ represent extracellular and intracellular concentrations, respectively, of RA; RA_signal_ represents the strength of the RA signal (which may or may not vary linearly with [RA]_in_, in accordance with exponent *n*); and [Cyp] represents the intracellular concentration of Cyp26a1. *D* is the effective extracellular diffusion coefficient of RA; *V*(*x*) is the rate of production of RA at position *x*, typically taken to be a constant in a fixed posterior domain and zero elsewhere; *k*_p_ is a first-order permeability coefficient that lumps together the processes of RA dissociation from protein, association with and diffusion through the plasma membrane, and reassociation with protein on the other side of the membrane; and *k*_deg_ is a degradation constant for intracellular RA. Effects of [RA]_in_ and Fgf on *cyp26a1* expression are captured through multiplication of the parameter *k*_deg_ by the expression containing γ, *f*_0_, λ, and *x*_f_. Here γ is sensitivity to RA_signal_-mediated feedback; *f*_0_ represents the sensitivity of feedback to Fgf; λ is the length constant of the Fgf gradient (the inverse of the distance over which it drops to *e* ^−1^ of its initial value); and *x*_f_ is the location along the Fgf gradient where [Fgf] = *f*_0_. The parameter β is introduced to allow the effective rate constant for flux of RA out of the extracellular space to be higher than for flux into the intracellular space (to capture the fact that some endogenous RA should leave the extracellular space entirely, e.g., by diffusing out of the embryo, or into long-term capture in yolk).

The *x*-axis is taken to represent the A–P axis of the gastrula-stage embryo, with *x* = 0 corresponding to the posterior border of an anterior domain of high *cyp26a1* expression (approximately the r2/r3 boundary), and *x* = *x*_f_ at the posterior margin. The boundary of a second domain of high *cyp26a1* expression is placed 40 μm from the posterior margin. The value of [Cyp] in both of these domains is fixed at *k*_max_. A no-flux boundary condition is placed at *x* = −200 (to represent the fact that the problem could be viewed as symmetrical about the anterior-most point of the embryo). At the posterior margin, it was assumed that extracellular RA could only leave the embryo by diffusing through cells, so a “leaky” posterior boundary condition was used, in which the parameter *k*_p_ captured the rate constant of leakage.

Steady-state solutions to the equations were obtained numerically using Mathematica software by solving the time-dependent equations for sufficiently long times that results no longer changed significantly (typically after a few hours). In some cases, steady-state calculations were done directly using a Matlab boundary value ODE Solver. Parameter values were manually explored over several orders of magnitude. In all panels of Figure 7, parameter values were *D* = 18 μm^2^sec^−1^, *n* = 2, λ = 0.019 μm^−1^, *k*_p_ = 10^−4^sec^−1^, and γ = 2.5 × 10^−5^. (Other parameter values are given in the figure legend.) The choice of modest cooperativity in signaling (*n* = 2) acknowledges the intrinsic capabilities of nuclear hormone receptors [60], but is not, in fact, required to produce the qualitative findings of this study. It should be noted that the length scales of the RA gradients produced in Figure 7 are determined largely by the length scale of the Fgf gradient (1/λ), which was chosen arbitrarily, and the diffusion coefficient *D* of protein-bound RA, which is not known accurately. Accordingly the micrometer values on the abscissa should be taken as essentially arbitrary and easily adjustable to be several fold smaller or larger through modest changes in these parameter values.

## Supporting Information

Figure S1 Induction of *rare:yfp* Reporter at Gastrula Stage (A–C) Implantation of a bead soaked in 10 mM RA at 8 hpf induces YFP expression after 2 h. Single confocal sections show nuclear YFP expression (A) and cells labeled with BODIPY-TR (B). (C) Merged image. dashed circle indicates the position of the implanted bead.
(5.7 MB TIF)

Figure S2 Response of *cyp26a1* to Global RA Treatment (A–D) *cyp26a1* is up-regulated in a concentration-dependent manner that also depends on a cell's position in the embryo (compare [B], [C], and [D] to [A]).
(3.1 MB TIF)

Figure S3 Global RA Treatments Rescue Hindbrain Segmentation over a Wide Range of Concentrations Embryos were treated at 4 hpf with DEAB either with or without a low concentration of RA between 0.625–20 nM, fixed at 19 hpf, and pattern was assessed by in situ hybridization for *hoxd4a* (blue; r6/7 boundary), *krox-20* (*egr2b*, red; r3 and r5), and *pax2a* (red; midbrain–hindbrain boundary).
(A) Wild-type expression.
(B) DEAB-treated embryos show loss of r5–r7.
(C–F) Concentrations from 0.625–5 nM rescue wild-type patterning to the same extent.
(G–H) Higher concentrations produce anterior defects.
(9.5 MB TIF)

### Accession Numbers

The GenBank (http://www.ncbi.nlm.nih.gov/Genbank) accession numbers for the genes discussed in this paper are *aldh1a2* (NM_131850), *cyp26a1* (NM_131146), *cyp26b1* (NM_212666), *cyp26c1* (NM_001029951), *hoxb1b* (NM_131142) *hoxb4* (NM_131118), *hoxb5a* (NM_131101), *hoxd4a* (NM_130757), *krox-20/egr2b* (NM_130997), and *pax2a* (NM_131184).

The ZFIN (Zebrafish Model Organism database; http://www.zfin.org) ID numbers are *aldh1a2* (ZDB-GENE-011010–3), *cyp26a1* (ZDB-GENE-990415–44), *cyp26b1* (ZDB-GENE-990415–44), *cyp26c1* (ZDB-GENE-050714–2), *hoxb1b* (ZDB-GENE-980526–290), *hoxb4* (ZDB-GENE-990415–105), *hoxb5a* (ZDB-GENE-980526–70), *hoxd4a* (ZDB-GENE-980526–214) *krox-20/egr2b* (ZDB-GENE-980526–283), and *pax2a* (ZDB-GENE-990415–8).

## Abbreviations

A–P: anterior–posterior
Fgf: fibroblast growth factor
hpf: hours post-fertilization
MO: Morpholino
RA: retinoic acid
RARE: retinoic acid response element
YFP: yellow fluorescent protein

## References

[pbio-0050304-b001] Cox WG, Hemmati-Brivanlou A (1995). Caudalization of neural fate by tissue recombination and bFGF. Development.

[pbio-0050304-b002] Lamb TM, Harland RM (1995). Fibroblast growth factor is a direct neural inducer, which combined with noggin generates anterior-posterior neural pattern. Development.

[pbio-0050304-b003] McGrew LL, Lai CJ, Moon RT (1995). Specification of the anteroposterior neural axis through synergistic interaction of the Wnt signaling cascade with noggin and follistatin. Dev Biol.

[pbio-0050304-b004] Koshida S, Shinya M, Mizuno T, Kuroiwa A, Takeda H (1998). Initial anteroposterior pattern of the zebrafish central nervous system is determined by differential competence of the epiblast. Development.

[pbio-0050304-b005] Kiecker C, Niehrs C (2001). A morphogen gradient of Wnt/beta-catenin signalling regulates anteroposterior neural patterning in Xenopus. Development.

[pbio-0050304-b006] Kudoh T, Wilson SW, Dawid IB (2002). Distinct roles for Fgf, Wnt and retinoic acid in posteriorizing the neural ectoderm. Development.

[pbio-0050304-b007] Scholpp S, Brand M (2004). Endocytosis controls spreading and effective signaling range of Fgf8 protein. Curr Biol.

[pbio-0050304-b008] Hernandez RE, Putzke AP, Myers JP, Margaretha L, Moens CB (2007). Cyp26 enzymes generate the retinoic acid response pattern necessary for hindbrain development. Development.

[pbio-0050304-b009] Niederreither K, Vermot J, Schuhbaur B, Chambon P, Dolle P (2000). Retinoic acid synthesis and hindbrain patterning in the mouse embryo. Development.

[pbio-0050304-b010] White JC, Highland M, Kaiser M, Clagett-Dame M (2000). Vitamin A deficiency results in the dose-dependent acquisition of anterior character and shortening of the caudal hindbrain of the rat embryo. Dev Biol.

[pbio-0050304-b011] Begemann G, Schilling TF, Rauch GJ, Geisler R, Ingham PW (2001). The zebrafish *neckless* mutation reveals a requirement for *raldh2* in mesodermal signals that pattern the hindbrain. Development.

[pbio-0050304-b012] Maves L, Kimmel CB (2005). Dynamic and sequential patterning of the zebrafish posterior hindbrain by retinoic acid. Dev Biol.

[pbio-0050304-b013] Molotkova N, Molotkov A, Sirbu IO, Duester G (2005). Requirement of mesodermal retinoic acid generated by Raldh2 for posterior neural transformation. Mech Dev.

[pbio-0050304-b014] Begemann G, Marx M, Mebus K, Meyer A, Bastmeyer M (2004). Beyond the *neckless* phenotype: influence of reduced retinoic acid signaling on motor neuron development in the zebrafish hindbrain. Dev Biol.

[pbio-0050304-b015] Gale E, Zile M, Maden M (1999). Hindbrain respecification in the retinoid-deficient quail. Mech Dev.

[pbio-0050304-b016] Maden M, Gale E, Kostetskii I, Zile M (1996). Vitamin A-deficient quail embryos have half a hindbrain and other neural defects. Curr Biol.

[pbio-0050304-b017] Dupe V, Lumsden A (2001). Hindbrain patterning involves graded responses to retinoic acid signalling. Development.

[pbio-0050304-b018] Durston AJ, Timmermans JP, Hage WJ, Hendriks HF, de Vries NJ (1989). Retinoic acid causes an anteroposterior transformation in the developing central nervous system. Nature.

[pbio-0050304-b019] Sive HL, Draper BW, Harland RM, Weintraub H (1990). Identification of a retinoic acid-sensitive period during primary axis formation in Xenopus laevis. Genes Dev.

[pbio-0050304-b020] Marshall H, Nonchev S, Sham MH, Muchamore I, Lumsden A (1992). Retinoic acid alters hindbrain Hox code and induces transformation of rhombomeres 2/3 into a 4/5 identity. Nature.

[pbio-0050304-b021] Godsave SF, Koster CH, Getahun A, Mathu M, Hooiveld M (1998). Graded retinoid responses in the developing hindbrain. Dev Dyn.

[pbio-0050304-b022] Abu-Abed S, Dolle P, Metzger D, Beckett B, Chambon P (2001). The retinoic acid-metabolizing enzyme, CYP26A1, is essential for normal hindbrain patterning, vertebral identity, and development of posterior structures. Genes Dev.

[pbio-0050304-b023] Sakai Y, Meno C, Fujii H, Nishino J, Shiratori H (2001). The retinoic acid-inactivating enzyme CYP26 is essential for establishing an uneven distribution of retinoic acid along the anterio-posterior axis within the mouse embryo. Genes Dev.

[pbio-0050304-b024] Emoto Y, Wada H, Okamoto H, Kudo A, Imai Y (2005). Retinoic acid-metabolizing enzyme Cyp26a1 is essential for determining territories of hindbrain and spinal cord in zebrafish. Dev Biol.

[pbio-0050304-b025] Sirbu IO, Gresh L, Barra J, Duester G (2005). Shifting boundaries of retinoic acid activity control hindbrain segmental gene expression. Development.

[pbio-0050304-b026] Eichele G, Thaller C (1987). Characterization of concentration gradients of a morphogenetically active retinoid in the chick limb bud. J Cell Biol.

[pbio-0050304-b027] Wolpert L (1989). Positional information revisited. Development.

[pbio-0050304-b028] Lumsden A, Krumlauf R (1996). Patterning the vertebrate neuraxis. Science.

[pbio-0050304-b029] Marshall H, Morrison A, Studer M, Popperl HRK (1996). Retinoids and Hox genes. FASEB J.

[pbio-0050304-b030] Perz-Edwards A, Hardison NL, Linney E (2001). Retinoic acid-mediated gene expression in transgenic reporter zebrafish. Dev Biol.

[pbio-0050304-b031] Prince VE, Joly L, Ekker M, Ho RK (1998). Zebrafish *hox* genes: genomic organization and modified colinear expression patterns in the trunk. Development.

[pbio-0050304-b032] Gould A, Itasaki N, Krumlauf R (1998). Initiation of rhombomeric *Hoxb4* expression requires induction by somites and a retinoid pathway. Neuron.

[pbio-0050304-b033] Oosterveen T, Niederreither K, Dolle P, Chambon P, Meijlink F (2003). Retinoids regulate the anterior expression boundaries of 5' Hoxb genes in posterior hindbrain. EMBO J.

[pbio-0050304-b034] Russo JE (1997). Inhibition of mouse and human class 1 aldehyde dehydrogenase by 4-(N,N-dialkylamino)benzaldehyde compounds. Adv Exp Med Biol.

[pbio-0050304-b035] Russo JE, Hauguitz D, Hilton J (1988). Inhibition of mouse cytosolic aldehyde dehydrogenase by 4-(diethylamino)benzaldehyde. Biochem Pharmacol.

[pbio-0050304-b036] Reijntjes S, Blentic A, Gale E, Maden M (2005). The control of morphogen signalling: regulation of the synthesis and catabolism of retinoic acid in the developing embryo. Dev Biol.

[pbio-0050304-b037] Dobbs-McAuliffe B, Zhao Q, Linney E (2004). Feedback mechanisms regulate retinoic acid production and degradation in the zebrafish embryo. Mech Dev.

[pbio-0050304-b038] Tsukada M, Schroder M, Seltmann H, Orfanos CE, Zouboulis CC (2002). High albumin levels restrict the kinetics of 13-cis retinoic acid uptake and intracellular isomerization to all-trans retinoic acid and inhibit its anti-proliferative effect on SZ95 sebocytes. J Invest Dermatol.

[pbio-0050304-b039] Eldar A, Rosin D, Shilo BZ, Barkai N (2003). Self-enhanced ligand degradation underlies robustness of morphogen gradients. Dev Cell.

[pbio-0050304-b040] Lander AD, Nie Q, Wan FY (2002). Do morphogen gradients arise by diffusion?. Dev Cell.

[pbio-0050304-b041] Lander AD, Nie Q, Vargas B, Wan FY (2005). Aggregation of a distributed source in morphogen gradient formation. SIAM Stud Appl Math.

[pbio-0050304-b042] Wolpert L (1971). Positional information and pattern formation. Curr Top Dev Biol.

[pbio-0050304-b043] Thaller C, Eichele G (1996). Retinoid signaling in vertebrate limb development. Ann N Y Acad Sci.

[pbio-0050304-b044] Moss JB, Xavier-Neto J, Shapiro MD, Nayeem SM, McCaffery P (1998). Dynamic patterns of retinoic acid synthesis and response in the developing mammalian heart. Dev Biol.

[pbio-0050304-b045] Tickle C (1999). Morphogen gradients in vertebrate limb development. Semin Cell Dev Biol.

[pbio-0050304-b046] Hochgreb T, Linhares VL, Menezes DC, Sampaio AC, Yan CY (2003). A caudorostral wave of RALDH2 conveys anteroposterior information to the cardiac field. Development.

[pbio-0050304-b047] Yashiro K, Zhao X, Uehara M, Yamashita K, Nishijima M (2004). Regulation of retinoic acid distribution is required for proximodistal patterning and outgrowth of the developing mouse limb. Dev Cell.

[pbio-0050304-b048] Nutt SL, Dingwell KS, Holt CE, Amaya E (2001). Xenopus Sprouty2 inhibits FGF-mediated gastrulation movements but does not affect mesoderm induction and patterning. Genes Dev.

[pbio-0050304-b049] Chung HA, Hyodo-Miura J, Nagamune T, Ueno N (2005). FGF signal regulates gastrulation cell movements and morphology through its target NRH. Dev Biol.

[pbio-0050304-b050] Adams J (1993). Structure-activity and dose-response relationships in the neural and behavioral teratogenesis of retinoids. Neurotoxicol Teratol.

[pbio-0050304-b051] Shiotsugu J, Katsuyama Y, Arima K, Baxter A, Koide T (2004). Multiple points of interaction between retinoic acid and FGF signaling during embryonic axis formation. Development.

[pbio-0050304-b052] Bergmann S, Sandler O, Sberro H, Shnider S, Schejter E (2007). Pre-steady-state decoding of the Bicoid morphogen gradient. PLoS Biol.

[pbio-0050304-b053] Goldbeter A, Gonze D, Pourquie O (2007). Sharp developmental thresholds defined through bistability by antagonistic gradients of retinoic acid and FGF signaling. Dev Dyn.

[pbio-0050304-b054] Baker RE, Maini PK (2007). Travelling gradients in interacting morphogen systems. Math Biosci.

[pbio-0050304-b055] Marletaz F, Holland LZ, Laudet V, Schubert M (2006). Retinoic acid signaling and the evolution of chordates. Int J Biol Sci.

[pbio-0050304-b056] Szanto A, Narkar V, Shen Q, Uray IP, Davies PJ (2004). Retinoid X receptors: X-ploring their (patho)physiological functions. Cell Death Differ.

[pbio-0050304-b057] King-Jones K, Horner MA, Lam G, Thummel CS (2006). The DHR96 nuclear receptor regulates xenobiotic responses in Drosophila. Cell Metab.

[pbio-0050304-b058] Niwa R, Matsuda T, Yoshiyama T, Namiki T, Mita K (2004). CYP306A1, a cytochrome P450 enzyme, is essential for ecdysteroid biosynthesis in the prothoracic glands of Bombyx and Drosophila. J Biol Chem.

[pbio-0050304-b059] Bogwitz MR, Chung H, Magoc L, Rigby S, Wong W (2005). Cyp12a4 confers lufenuron resistance in a natural population of Drosophila melanogaster. Proc Natl Acad Sci U S A.

[pbio-0050304-b060] Kerszberg M (1996). Accurate reading of morphogen concentrations by nuclear receptors: a formal model of complex transduction pathways. J Theor Biol.

[pbio-0050304-b061] Kimmel CB, Ballard WW, Kimmel SR, Ullmann B, Schilling TF (1995). Stages of embryonic development of the zebrafish. Dev Dyn.

[pbio-0050304-b062] Thisse C, Thisse B, Schilling TF, Postlethwait JH (1993). Structure of the zebrafish *snail1* gene and its expression in wild-type, *spadetail* and *no tail* mutant embryos. Development.

[pbio-0050304-b063] Kawakami Y, Raya A, Raya RM, Rodriguez-Esteban C, Belmonte JC (2005). Retinoic acid signalling links left-right asymmetric patterning and bilaterally symmetric somitogenesis in the zebrafish embryo. Nature.

[pbio-0050304-b064] McClintock JM, Carlson R, Mann DM, Prince VE (2001). Consequences of Hox gene duplication in the vertebrates: an investigation of the zebrafish Hox paralogue group 1 genes. Development.

[pbio-0050304-b065] Oxtoby E, Jowett T (1993). Cloning of the zebrafish *krox-20* gene (krx-20) and its expression during hindbrain development. Nucleic Acids Res.

[pbio-0050304-b066] Krauss S, Johansen T, Korzh V, Fjose A (1991). Expression of the zebrafish paired box gene pax[zf-b] during early neurogenesis. Development.

[pbio-0050304-b067] von Dassow G, Meir E, Munro EM, Odell GM (2000). The segment polarity network is a robust developmental module. Nature.

[pbio-0050304-b068] Eldar A, Dorfman R, Weiss D, Ashe H, Shilo BZ (2002). Robustness of the BMP morphogen gradient in Drosophila embryonic patterning. Nature.

